# Supplementary figures and images for: Triadic relationships between pasture exposure, gastrointestinal parasites, and hindgut microbiomes in grazing lambs
Source: PLoS One. 2025 Nov 17;20(11):e0337086. doi: 10.1371/journal.pone.0337086 (PMC12622837; doi:10.1371/journal.pone.0337086)

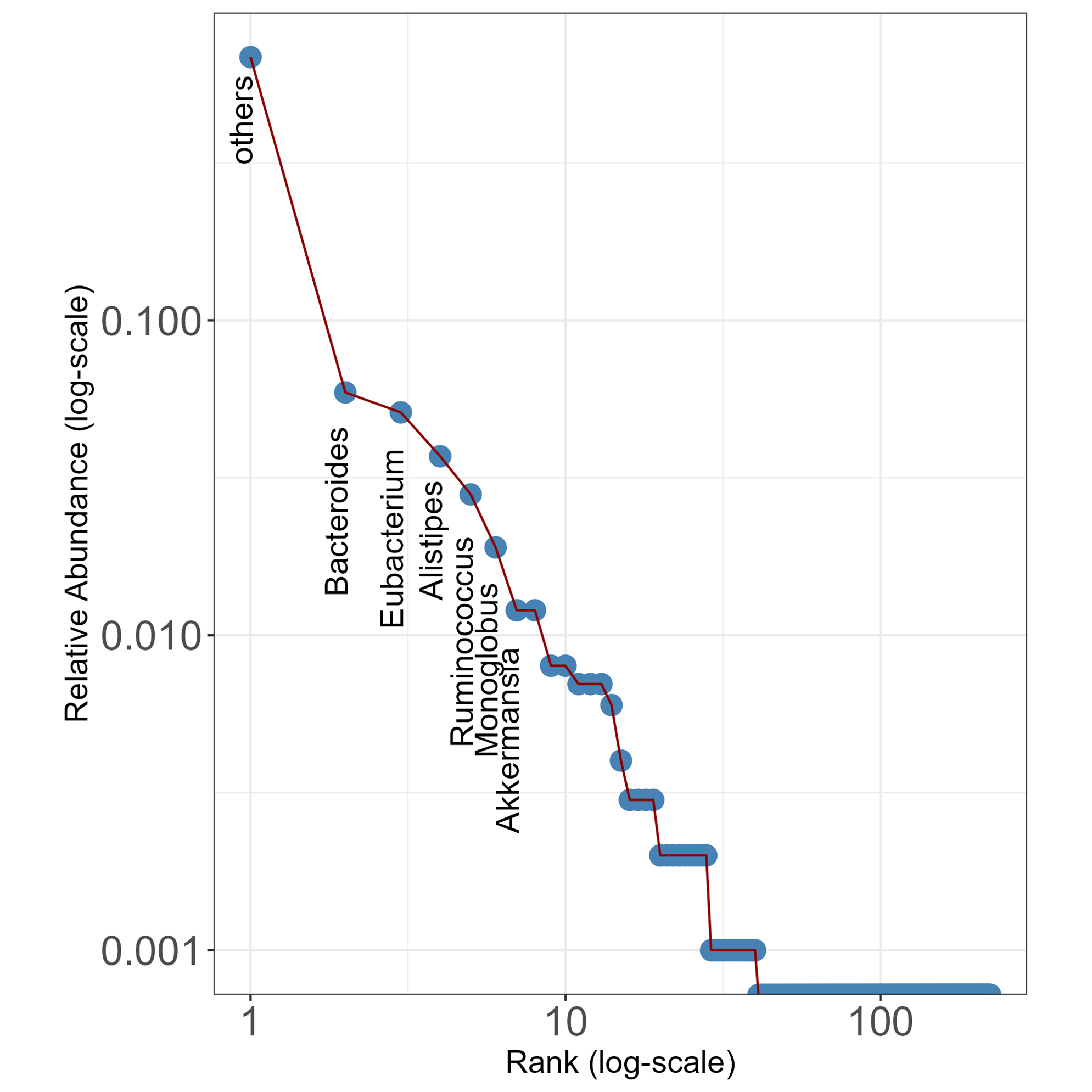

Supplement: S1 Fig — Rank–abundance distribution of bacterial genera based on the total number of reads from all 60 samples. Genera are ranked by their total read counts, and their relative abundances are displayed on a logarithmic scale. Labels identify the six most abundant genera and the aggregated “other” group, which includes all ASVs not assigned to a genus level. (TIFF) [file pone.0337086.s001.tiff]

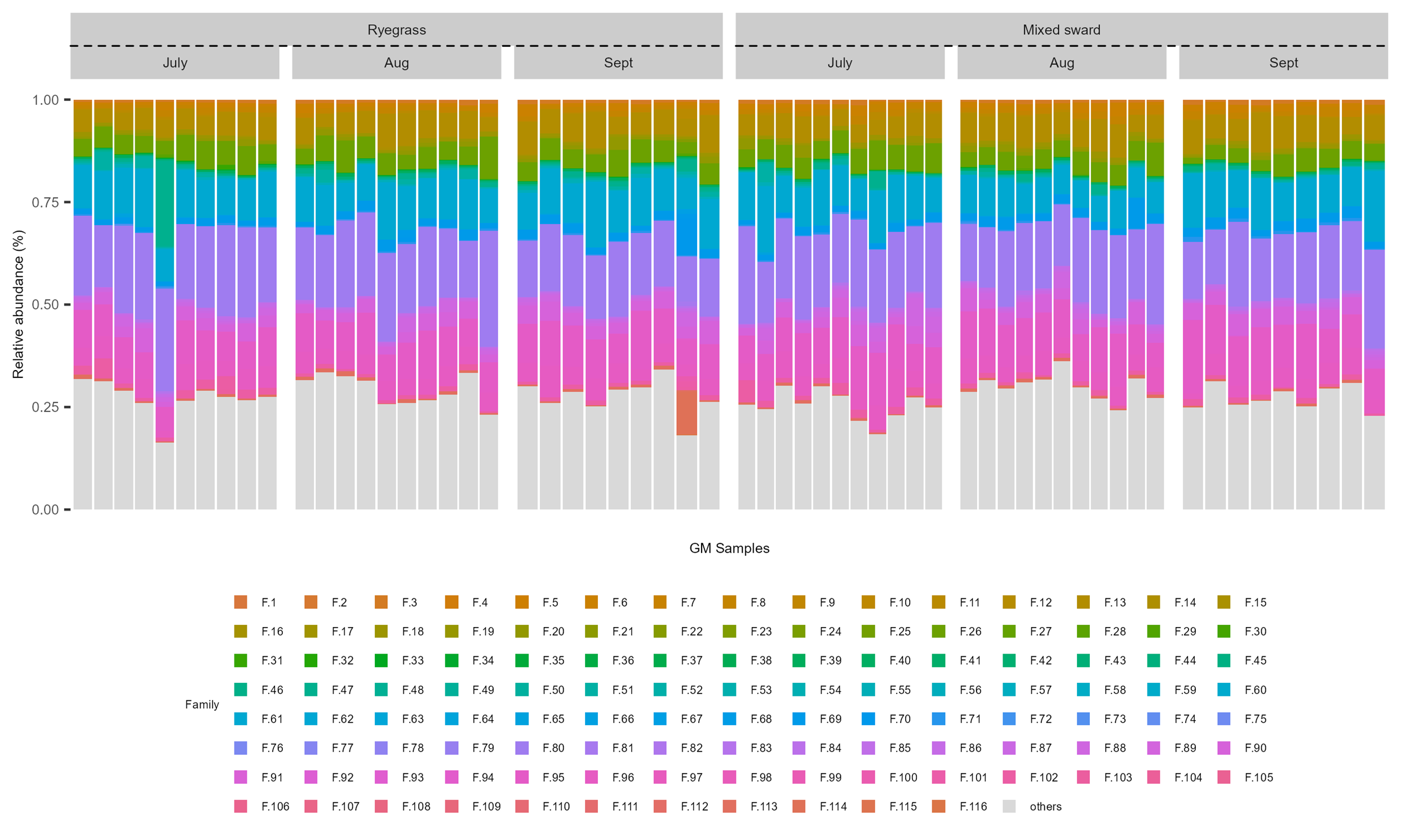

Supplement: S2 Fig — Gut microbial (GM) composition in lambs grazing on two sward types (ryegrass and mixed sward) over three consecutive months. Stacked bar plots show the relative abundance of 16S rRNA gene sequences assigned to different bacterial families. A total of 116 identifiable families were detected, including Oscillospiraceae, Bacteroidaceae, Rikenellaceae, Campylobacteraceae, Acidaminococcaceae, Enterobacteriaceae, Prevotellaceae, Lachnospiraceae, Akkermansiaceae, Ruminococcaceae, Christensenellaceae, Butyricicoccaceae, Pirellulaceae, Planococcaceae, Monoglobaceae, Desulfovibrionaceae, Anaerovoracaceae, Spirochaetaceae, Moraxellaceae, Weeksellaceae, Hungateiclostridiaceae, Peptostreptococcaceae, Fibrobacteraceae, Defluviitaleaceae, Erysipelotrichaceae, Peptococcaceae, Helicobacteraceae, Bacillaceae, Victivallaceae, Paludibacteraceae, Bifidobacteriaceae, Barnesiellaceae, Clostridiaceae, Marinifilaceae, Flavobacteriaceae, Muribaculaceae, Eggerthellaceae, Myxococcaceae, Atopobiaceae, Elusimicrobiaceae, Marinilabiliaceae, Deferribacteraceae, Streptococcaceae, Mycoplasmataceae, Sphingomonadaceae, Acholeplasmataceae, Acetobacteraceae, Tannerellaceae, Sphingobacteriaceae, Oxalobacteraceae, Saccharimonadaceae, Micrococcaceae, Corynebacteriaceae, Staphylococcaceae, Caulobacteraceae, Oligosphaeraceae, Aerococcaceae, Puniceicoccaceae, Enterococcaceae, Carnobacteriaceae, Nocardioidaceae, Hymenobacteraceae, Comamonadaceae, Endomicrobiaceae, Anaerolineaceae, Rhodobacteraceae, Dysgonomonadaceae, Microbacteriaceae, Rhizobiaceae, Pseudomonadaceae, Xanthomonadaceae, Selenomonadaceae, Beijerinckiaceae, Caldicoprobacteraceae, Geodermatophilaceae, Eubacteriaceae, Dermabacteraceae, Paenibacillaceae, Sutterellaceae, Dietziaceae, Succinivibrionaceae, Nitrosomonadaceae, Porphyromonadaceae, Hydrogenophilaceae, Veillonellaceae, Chitinophagaceae, Intrasporangiaceae, Terasakiellaceae, Xanthobacteraceae, Devosiaceae, Nocardiaceae, Cellvibrionaceae, Lactobacillaceae, Chlamydiaceae, Synergistaceae, Coriob [file pone.0337086.s002.tiff]
