# Supplementary material for: Triadic relationships between pasture exposure, gastrointestinal parasites, and hindgut microbiomes in grazing lambs
Source: PLoS One. 2025 Nov 17;20(11):e0337086. doi: 10.1371/journal.pone.0337086 (PMC12622837; doi:10.1371/journal.pone.0337086)
Supplement: S1 Table — The table lists significant effects of predictors on the relative abundance of the most common bacterial genera, as estimated from a generalized linear latent variable model (GLLVM) with a negative binomial distribution. ‘Significant’ effects are defined as those whose 95% confidence intervals of the coefficient estimates do not overlap zero. Columns indicate the predictor (treatment: month, sward type, or parasite load), genus, family, phylum, and the model estimate (with 95% confidence interval in parentheses). Positive estimates indicate an increase in relative abundance relative to the model baseline, and negative estimates indicate a decrease. (DOCX) [file pone.0337086.s003.docx]

**S1 Table. Significant predictor effects on the differential abundance of common bacterial genera in lamb gut microbiomes.** The table lists significant effects of predictors on the relative abundance of the most common bacterial genera, as estimated from a generalized linear latent variable model (GLLVM) with a negative binomial distribution. ‘Significant’ effects are defined as those whose 95% confidence intervals of the coefficient estimates do not overlap zero. Columns indicate the predictor (treatment: month, sward type, or parasite load), genus, family, phylum, and the model estimate (with 95% confidence interval in parentheses). Positive estimates indicate an increase in relative abundance relative to the model baseline, and negative estimates indicate a decrease.

| **Treatment** | **Genus** | **Family** | **Phylum** | **Estimate** |
| --- | --- | --- | --- | --- |
| July | Acetitomaculum | Lachnospiraceae | Firmicutes | -1.78 (-3.29 – -0.28) |
| Sept | Acetitomaculum | Lachnospiraceae | Firmicutes | -0.7 (-1.33 – -0.08) |
| Strongyles | Acetitomaculum | Lachnospiraceae | Firmicutes | -0.77 (-1.45 – -0.09) |
| July | Acetobacter | Acetobacteraceae | Proteobacteria | -1.51 (-2.45 – -0.58) |
| Mixed sward | Alistipes | Rikenellaceae | Bacteroidota | 0.29 (0.03 – 0.55) |
| July | Alistipes | Rikenellaceae | Bacteroidota | -0.43 (-0.68 - -0.18) |
| July | Anaerosporobacter | Lachnospiraceae | Firmicutes | 1.75 (0.25 – 3.26) |
| Sept | Anaerosporobacter | Lachnospiraceae | Firmicutes | 1.95 (1.01 – 2.88) |
| Nematodirus | Anaerosporobacter | Lachnospiraceae | Firmicutes | 0.93 (0.22 – 1.65) |
| July | Anaerovorax | Anaerovoracaceae | Firmicutes | -0.62 (-1.09 - -0.14) |
| Strongyles | Anaerovorax | Anaerovoracaceae | Firmicutes | -0.18 (-0.32 – -0.04) |
| July | Bacteroides | Bacteroidaceae | Bacteroidota | -0.43 (-0.6 – -0.26) |
| Sept | Bacteroides | Bacteroidaceae | Bacteroidota | -0.12 (-0.21 – -0.03) |
| Nematodirus | Bacteroides | Bacteroidaceae | Bacteroidota | -0.11 (-0.2 – -0.02) |
| July | Butyrivibrio | Lachnospiraceae | Firmicutes | 2.11 (0.34 – 3.88) |
| Sept | Butyrivibrio | Lachnospiraceae | Firmicutes | 1.79 (0.9 – 2.67) |
| Strongyles | Butyrivibrio | Lachnospiraceae | Firmicutes | 0.81 (0.25 – 1.36) |
| Nematodirus | Candidatus Saccharimonas | Saccharimonadaceae | others | -1.32 (-2.57 - -0.08) |
| Strongyles | Candidatus Saccharimonas | Saccharimonadaceae | others | 0.92 (0.75 – 1.09) |
| Mixed sward | Candidatus_Soleaferrea | Ruminococcaceae | Firmicutes | 0.26 (0.05 – 0.48) |
| July | Candidatus_Soleaferrea | Ruminococcaceae | Firmicutes | -0.31 (-0.52 – -0.11) |
| Sept | Candidatus_Soleaferrea | Ruminococcaceae | Firmicutes | -0.21 (-0.32 – -0.1) |
| Nematodirus | Candidatus_Soleaferrea | Ruminococcaceae | Firmicutes | -0.16 (-0.27 – -0.05) |
| Sept | Clostridioides | Peptostreptococcaceae | Firmicutes | 0.59 (0.08 - 1.1) |
| July | Colidextribacter | Oscillospiraceae | Firmicutes | -0.51 (-0.94 – -0.09) |
| Sept | Colidextribacter | Oscillospiraceae | Firmicutes | -0.39 (-0.61 – -0.17) |
| Nematodirus | Colidextribacter | Oscillospiraceae | Firmicutes | -0.28 (-0.5 – -0.05) |
| Sept | Coprococcus | Lachnospiraceae | Firmicutes | -0.5 (-0.78 – -0.22) |
| Sept | Desulfovibrio | Desulfovibrionaceae | Desulfobacterota | -0.52 (-0.88 – -0.15) |
| Nematodirus | Desulfovibrio | Desulfovibrionaceae | Desulfobacterota | -0.32 (-0.61 – -0.03) |
| July | Elusimicrobium | Elusimicrobiaceae | Elusimicrobiota | -1.07 (-1.91 – -0.23) |
| July | Escherichia-Shigella | Enterobacteriaceae | Proteobacteria | 1.23 (0.28 – 2.18) |
| Strongyles | Escherichia-Shigella | Enterobacteriaceae | Proteobacteria | 0.38 (0.26 – 0.5) |
| July | Eubacterium | others | Firmicutes | -0.22 (-0.36 – -0.08) |
| Sept | Eubacterium | others | Firmicutes | -0.19 (-0.27 – -0.11) |
| Strongyles | Helicobacter | Helicobacteraceae | Campilobacterota | -1.06 (-1.86 – -0.26) |
| July | Hydrogenoanaerobacterium | others | Firmicutes | -0.99 (-1.94 – -0.03) |
| July | Lysinibacillus | Planococcaceae | Firmicutes | 1.15 (0.35 – 1.96) |
| Nematodirus | Lysinibacillus | Planococcaceae | Firmicutes | 0.44 (0.03 – 0.86) |
| Strongyles | Lysinibacillus | Planococcaceae | Firmicutes | -0.26 (-0.44 – -0.09) |
| July | Mailhella | Desulfovibrionaceae | Desulfobacterota | -0.47 (-0.68 – -0.25) |
| Sept | Mailhella | Desulfovibrionaceae | Desulfobacterota | -0.23 (-0.35 – -0.12) |
| Sept | Mogibacterium | Anaerovoracaceae | Firmicutes | -0.22 (-0.36 – -0.07) |
| Mixed sward | Monoglobus | Monoglobaceae | Firmicutes | 0.27 (0.03 – 0.5) |
| July | Monoglobus | Monoglobaceae | Firmicutes | -0.58 (-0.8 – -0.36) |
| Sept | Monoglobus | Monoglobaceae | Firmicutes | -0.22 (-0.34 – -0.1) |
| Sept | Negativibacillus | Ruminococcaceae | Firmicutes | -2.39 (-3.78 – -0.99) |
| Nematodirus | Negativibacillus | Ruminococcaceae | Firmicutes | -2.01 (-3.33 – -0.7) |
| Strongyles | Olsenella | Atopobiaceae | Actinobacteriota | -0.85 (-1.09 – -0.61) |
| Mixed sward | Oscillibacter | Oscillospiraceae | Firmicutes | 0.66 (0.36 – 0.97) |
| July | Oscillibacter | Oscillospiraceae | Firmicutes | -0.59 (-0.89 - -0.3) |
| Sept | Oscillibacter | Oscillospiraceae | Firmicutes | -0.59 (-0.74 – -0.43) |
| Nematodirus | Oscillibacter | Oscillospiraceae | Firmicutes | -0.26 (-0.41 – -0.11) |
| July | Parabacteroides | Tannerellaceae | Bacteroidota | -1.38 (-2.67 – -0.08) |
| Sept | Parvibacter | Eggerthellaceae | Actinobacteriota | -0.64 (-1.08 – -0.19) |
| July | Phascolarctobacterium | Acidaminococcaceae | Firmicutes | -0.24 (-0.42 – -0.06) |
| Nematodirus | Romboutsia | Peptostreptococcaceae | Firmicutes | 0.31 (0.03 – 0.6) |
| Sept | Ruminococcus | Ruminococcaceae | Firmicutes | -0.32 (-0.47 – -0.18) |
| Mixed sward | Saccharofermentans | Hungateiclostridiaceae | Firmicutes | -1.11 (-1.65 – -0.56) |
| Sept | Solibacillus | Planococcaceae | Firmicutes | -1.11 (-1.52 – -0.7) |
| July | Streptococcus | Streptococcaceae | Firmicutes | 1.91 (0.29 – 3.53) |
| Sept | Streptococcus | Streptococcaceae | Firmicutes | -0.91 (-1.72 – -0.1) |
| Strongyles | Streptococcus | Streptococcaceae | Firmicutes | 0.36 (0.11 – 0.61) |
| July | Treponema | Spirochaetaceae | Spirochaetota | 0.58 (0.27 – 0.89) |
| Sept | Victivallis | Victivallaceae | Verrucomicrobiota | -0.41 (-0.82 – 0) |
