# Supplementary material for: Triadic relationships between pasture exposure, gastrointestinal parasites, and hindgut microbiomes in grazing lambs
Source: PLoS One. 2025 Nov 17;20(11):e0337086. doi: 10.1371/journal.pone.0337086 (PMC12622837; doi:10.1371/journal.pone.0337086)
Supplement: S2 Table — The table lists significant effects of predictors on the relative abundance of the most common bacterial families, estimated using a generalized linear latent variable model (GLLVM) with a negative binomial distribution. ‘Significant’ effects are defined as those whose 95% confidence intervals of the coefficient estimates do not overlap zero. Columns indicate the predictor (treatment: month, sward type, or parasite load), family, phylum, and the model estimate (with 95% confidence interval in parentheses). Positive estimates indicate an increase in relative abundance relative to the model baseline, while negative estimates indicate a decrease. (DOCX) [file pone.0337086.s004.docx]

**S2 Table. Significant predictor effects on the differential abundance of common bacterial families in lamb gut microbiomes.** The table lists significant effects of predictors on the relative abundance of the most common bacterial families, estimated using a generalized linear latent variable model (GLLVM) with a negative binomial distribution. ‘Significant’ effects are defined as those whose 95% confidence intervals of the coefficient estimates do not overlap zero. Columns indicate the predictor (treatment: month, sward type, or parasite load), family, phylum, and the model estimate (with 95% confidence interval in parentheses). Positive estimates indicate an increase in relative abundance relative to the model baseline, while negative estimates indicate a decrease.

| **Treatment** | **Family** | **Phylum** | **Estimate** |
| --- | --- | --- | --- |
| July | Acetobacteraceae | Proteobacteria | -1.17 (-2.05 – -0.29) |
| July | Acidaminococcaceae | Firmicutes | -0.18 (-0.36 – 0) |
| Mixed sward | Anaerovoracaceae | Firmicutes | 0.38 (0.19 – 0.56) |
| July | Anaerovoracaceae | Firmicutes | -0.23 (-0.41 – -0.05) |
| Nematodirus | Anaerovoracaceae | Firmicutes | -0.1 (-0.19 – -0.01) |
| Strongyle | Atopobiaceae | Actinobacteriota | -0.87 (-1.5 – -0.24) |
| July | Bacillaceae | Firmicutes | 1.16 (0.36 – 1.96) |
| Sept | Bacillaceae | Firmicutes | 0.69 (0.26 – 1.12) |
| Nematodirus | Bacillaceae | Firmicutes | 0.65 (0.24 – 1.06) |
| Strongyle | Bacillaceae | Firmicutes | -0.19 (-0.33 – -0.05) |
| July | Bacteroidaceae | Bacteroidota | -0.36 (-0.53 – -0.2) |
| Nematodirus | Bacteroidaceae | Bacteroidota | -0.1 (-0.19 – -0.02) |
| Mixed sward | Butyricicoccaceae | Firmicutes | 0.26 (0.05 – 0.47) |
| Sept | Butyricicoccaceae | Firmicutes | -0.16 (-0.26 – -0.05) |
| Sept | Christensenellaceae | Firmicutes | -0.25 (-0.35 – -0.15) |
| Nematodirus | Christensenellaceae | Firmicutes | -0.1 (-0.19 – 0) |
| Nematodirus | Clostridiaceae | Firmicutes | 0.44 (0.03 – 0.85) |
| Strongyle | Deferribacteraceae | Deferribacterota | 0.28 (0.11 – 0.45) |
| July | Desulfovibrionaceae | Desulfobacterota | -0.4 (-0.61 – -0.19) |
| Sept | Desulfovibrionaceae | Desulfobacterota | -0.17 (-0.28 – -0.06) |
| Mixed sward | Eggerthellaceae | Actinobacteriota | -0.56 (-0.9 – -0.23) |
| July | Elusimicrobiaceae | Elusimicrobiota | -1.01 (-1.84 – -0.19) |
| Sept | Elusimicrobiaceae | Elusimicrobiota | 0.44 (0.02 – 0.87) |
| Sept | Erysipelotrichaceae | Firmicutes | 0.2 (0.01 – 0.39) |
| Sept | Flavobacteriaceae | Bacteroidota | 0.47 (0.07 – 0.87) |
| Nematodirus | Flavobacteriaceae | Bacteroidota | 0.49 (0.07 – 0.91) |
| Strongyle | Helicobacteraceae | Campilobacterota | -1.14 (-1.39 – -0.89) |
| Mixed sward | Hungateiclostridiaceae | Firmicutes | -0.75 (-1.08 – -0.43) |
| Sept | Lachnospiraceae | Firmicutes | 0.1 (0.02 – 0.19) |
| Nematodirus | Lachnospiraceae | Firmicutes | -0.09 (-0.18 – -0.01) |
| Mixed sward | Monoglobaceae | Firmicutes | 0.25 (0.02 – 0.48) |
| July | Monoglobaceae | Firmicutes | -0.53 (-0.74 – -0.31) |
| Sept | Monoglobaceae | Firmicutes | -0.16 (-0.28 – -0.05) |
| Sept | Muribaculaceae | Bacteroidota | 0.57 (0.29 – 0.85) |
| Sept | Myxococcaceae | Myxococcota | -1.08 (-1.85 – -0.32) |
| Strongyle | Myxococcaceae | Myxococcota | -0.95 (-1.07 – -0.83) |
| Sept | Oscillospiraceae | Firmicutes | -0.22 (-0.29 – -0.15) |
| July | Paludibacteraceae | Bacteroidota | 1.21 (0.39 – 2.02) |
| Sept | Paludibacteraceae | Bacteroidota | 0.9 (0.45 – 1.35) |
| Mixed sward | Peptococcaceae | Firmicutes | 0.38 (0.1 – 0.66) |
| Sept | Peptostreptococcaceae | Firmicutes | 0.42 (0.14 – 0.7) |
| Nematodirus | Peptostreptococcaceae | Firmicutes | 0.33 (0.07 – 0.59) |
| Nematodirus | Planococcaceae | Firmicutes | 0.45 (0.05 – 0.86) |
| Strongyle | Planococcaceae | Firmicutes | -0.23 (-0.43 – -0.04) |
| Sept | Prevotellaceae | Bacteroidota | 0.16 (0.03 – 0.29) |
| Mixed sward | Rikenellaceae | Bacteroidota | 0.2 (0.02 – 0.38) |
| July | Rikenellaceae | Bacteroidota | -0.21 (-0.38 – -0.03) |
| Sept | Ruminococcaceae | Firmicutes | -0.15 (-0.25 – -0.04) |
| Nematodirus | Saccharimonadaceae | others | -1.54 (-2.61 – -0.46) |
| Strongyle | Saccharimonadaceae | others | 0.65 (0.41 – 0.89) |
| July | Spirochaetaceae | Spirochaetota | 0.66 (0.36 – 0.95) |
| July | Streptococcaceae | Firmicutes | 1.69 (0.15 – 3.24) |
| Sept | Streptococcaceae | Firmicutes | -0.95 (-1.7 – -0.2) |
| July | Victivallaceae | Verrucomicrobiota | -0.39 (-0.67 – -0.12) |
| Sept | Victivallaceae | Verrucomicrobiota | -0.46 (-0.6 – -0.31) |
